# Charles Bernard (Bernie) Carpenter, a giant of a mentor

**DOI:** 10.3389/frtra.2025.1631948

**Published:** 2025-06-11

**Authors:** Mohamed H. Sayegh

**Affiliations:** ^1^ American University of Beirut, Beirut, Lebanon; ^2^ Brigham and Women’s Hospital, Harvard Medical School, Boston, MA, United States

**Keywords:** transplantation, Harvard, Carpenter, Sayegh, mentor

During my nephrology fellowship interviews in 1986, I consistently received the advice that if I was genuinely interested in transplantation immunobiology, I should seek training with Bernie Carpenter at Brigham and Women's Hospital, Harvard Medical School. At the time, I interpreted this as a polite way of being turned down by the programs I visited. However, in 2005, during a celebration for my endowed chair at Harvard Medical School, Bernie introduced me by saying, “I knew MO before I met MO.” This connection was facilitated by Dr. William Braun from the Cleveland Clinic, who had trained me during my internal residency and recommended me to Bernie without reservation. I consider myself incredibly fortunate, particularly in my academic journey, as training under Bernie Carpenter was transformative. His unwavering commitment to prioritizing my interests over his own is a rare quality in academia, making my experience even more valuable.

Bernie was born on September 11, 1933, in Melrose, Massachusetts. He graduated summa cum laude from Dartmouth College in 1955, followed by Dartmouth Medical School in 1956, and earned his medical degree from Harvard Medical School in 1958. After completing his internal medicine residency at Cornell University Medical College and Bellevue Hospital in New York from 1958 to 1960, he served in the US Naval Medical Corps in Japan for two years. In 1962, he returned to Boston to train in nephrology at Peter Bent Brigham Hospital (now Brigham and Women's Hospital) under the guidance of Dr. John P. Merrill. In my humble opinion, I regard Merrill as the grandfather of transplantation medicine, with Bernie as a pivotal figure in its development. He played a crucial role in shaping the future of immunosuppression of kidney transplantation after the first successful kidney transplant by Nobel Laureate Dr. Joseph Murray at the Peter Bent Brigham in 1954 (1). While his academic contributions to transplantation medicine are well-documented and widely recognized (2, 3), I wish to focus on Bernie as a person—his humanity and mentorship.

There are many good mentors and even great ones, but Bernie stands out as a true giant in mentorship. His influence has shaped the careers of countless transplant physicians worldwide, a fact that is hard to overstate. During our time together starting in 1987, he consistently consulted me before allocating any of his grant funds. In 1997, just seven years after joining the faculty, Bernie took the initiative to approach the chair of medicine at Brigham and Women's Hospital, advocating for my appointment as the Director of Immunogenetics and Transplantation Research without me ever requesting it. Our bond resembled that of an academic father or older brother, always looking out for my best interests. After completing my fellowship in 1990, I was offered the Director of Medical Transplantation position, but Terry Strom, another of Bernie's mentees, advised me to focus on my academic career and go back to the research lab instead. After discussing it with Bernie, I chose to follow that advice, which ultimately shaped the trajectory of my career (Figure 1). I consider myself incredibly fortunate to have had such remarkable mentors guiding me.

**Figure 1:**
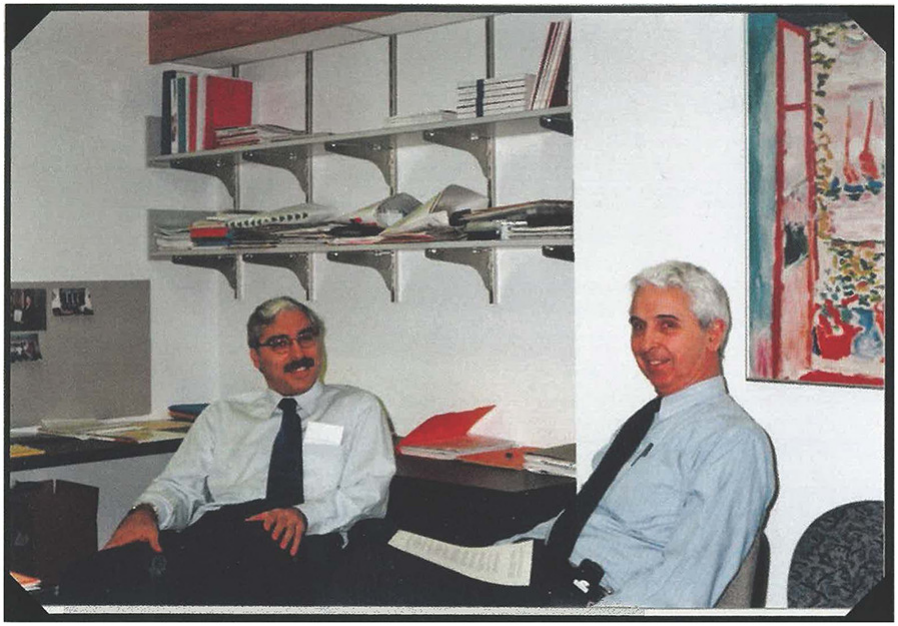
A picture of Dr.'s Sayegh and Carpenter in Dr. Sayegh's office.

What stands out about Bernie is his remarkable ability to remain calm and composed, never displaying anger or frustration. He approaches every challenge with the belief that a solution exists, and his legendary wisdom fosters an atmosphere of tranquility wherever he goes. Upon entering a room, he has an uncanny knack for diffusing tension and resolving issues, making him a truly humble presence. A notable example of this humility can be seen in the photograph of the renal division from the early 2000s, where Bernie is standing the last person in the back but clearly one of the greatest of pillars of academic medicine and transplantation (Figure 2).

**Figure 2:**
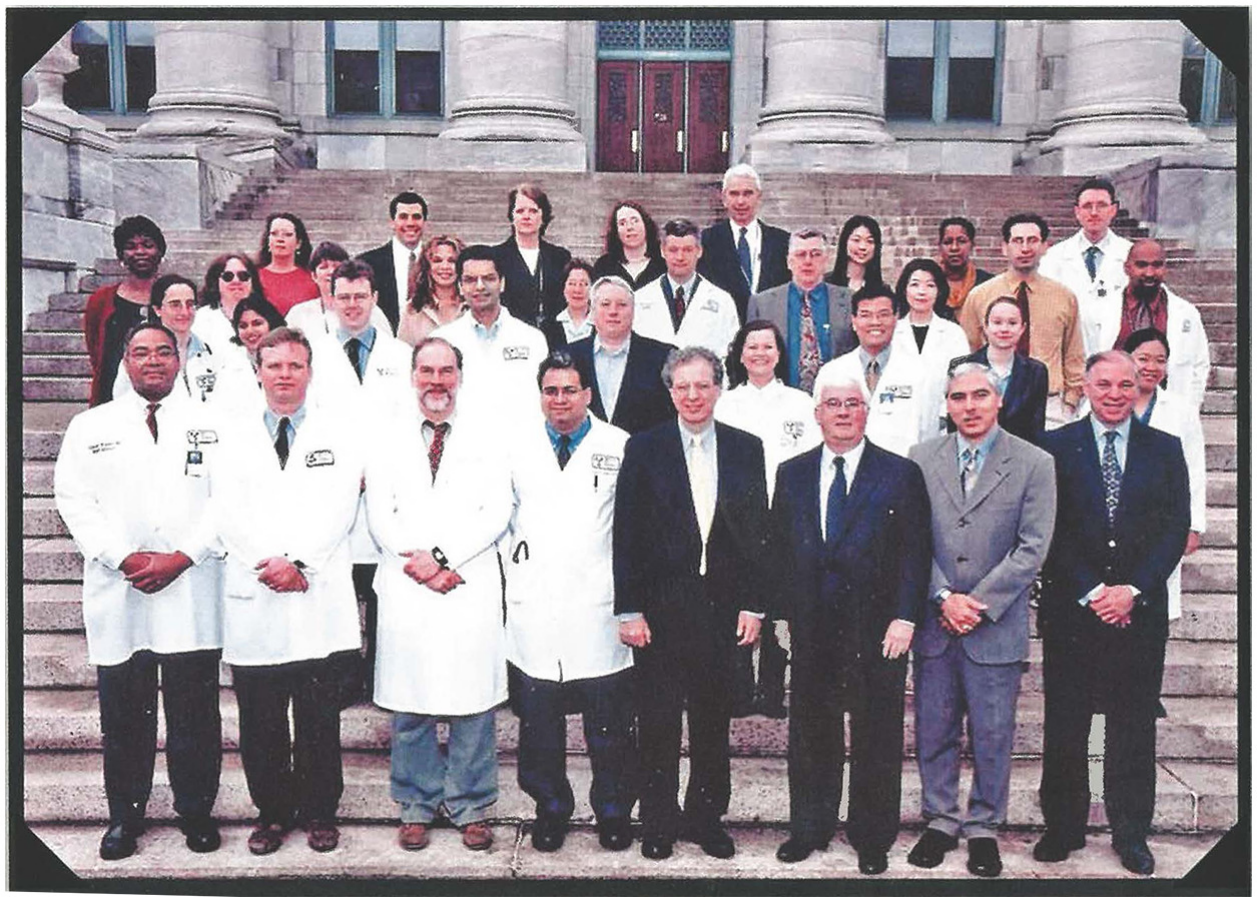
A picture of the Renal Division in front of the Peter Bent Hospital entrance.

The most poignant moment in our relationship occurred during the 2001 Past Presidents Dinner of the American Society of Transplantation. As I was concluding my term as President, past presidents, family, and friends were invited to share their thoughts about me. When it was Bernie's turn at the podium, he chose not to recount personal anecdotes but instead read a powerful poem by Robert Frost titled “How Hard it is to Keep From Being King When it is in You and the Situation.” The room fell silent, with many moved to tears by the depth of the words.

The most heartbreaking moment of my life occurred when Bernie walked into my office one day and posed a question, only to return later to ask the same thing again. Concerned, I turned to Jean Kutt, our senior laboratory technician at the time, to see if she had observed the same troubling signs, and she confirmed that she had. These instances marked the early indications of his struggle with Alzheimer's disease. Bernie officially retired in 2005 and sadly passed away on September 11, 2011, at the age of 78, leaving behind a legacy as a remarkable giant of a mentor.

## Data Availability

The original contributions presented in the study are included in the article/Supplementary Material, further inquiries can be directed to the corresponding author.
